# Smart double‐screening system of propagating male‐sterile lines for maize hybrid seed production

**DOI:** 10.1111/jipb.70331

**Published:** 2026-06-23

**Authors:** Ruidan Liu, Shengxi Hou, Hui Guo, Yang Cui, Weibin Song, Jie Yang, Hainan Zhao, Qiujie Liu, Jian Chen, Jinsheng Lai, Haiming Zhao

**Affiliations:** ^1^ State Key Laboratory of Maize Bio‐breeding, College of Agronomy and Biotechnology China Agricultural University Beijing 100193 China; ^2^ Sanya Institute of China Agricultural University Sanya 572025 China; ^3^ Frontiers Science Center for Molecular Design Breeding China Agricultural University Beijing 100193 China; ^4^ Institute of Crop Research Academy of Agricultural Sciences of Xinjiang Uyghur Autonomous Region Urumqi 830091 China

## Abstract

The Seed and Seedling Marker Method double‐screening system for maize hybrid seed production integrates *Ms45*‐mediated fertility restoration, *Mn1/Sh1*‐RNAi‐based seed selection, and *Lc*‐based seedling selection. It thus enables efficient, high‐purity propagation of male‐sterile lines by combining fertility maintenance with sequential visual selection at the seed and seedling stages.

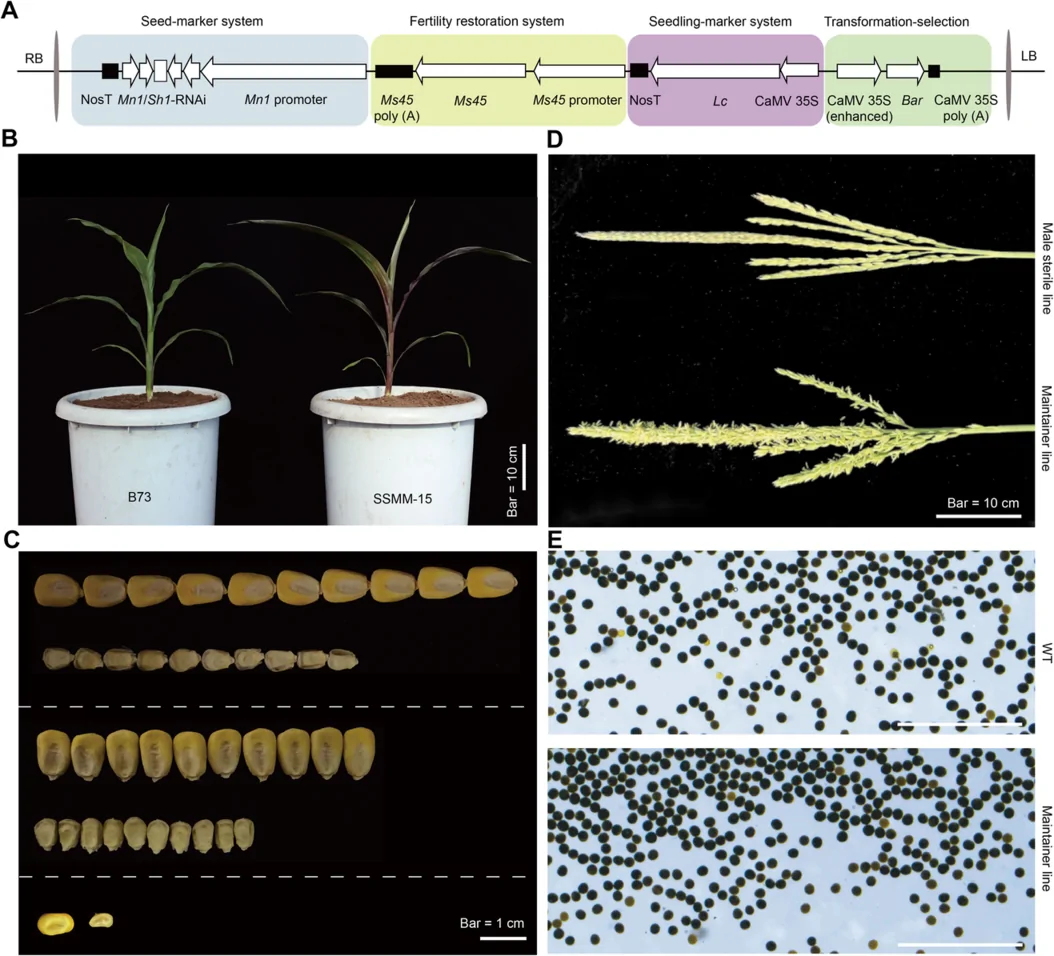

Maize is a monoecious, cross‐pollinated monocot. These traits facilitated the early exploitation of heterosis, with hybrid varieties typically outperforming inbred lines by 15%–50% in yield (Tester and Langridge, 2010). Maize hybrid production generally relies on manual or mechanical detasseling. Manual detasseling is labor‐intensive, inefficient, and costly, whereas mechanical detasseling requires flat fields and can damage maize leaves, thereby reducing yield. Therefore, using male‐sterile lines to produce maize hybrids could decrease production costs and improve seed purity (Chen and Liu, 2014).

Numerous nuclear male sterility (NMS) mutants have been identified in maize. Most of these mutants are controlled by recessive genes, are largely independent of genetic background, and do not require specific restorer lines, making NMS systems highly valuable for breeding. For example, Seed Production Technology (SPT) incorporates *Ms45*, a pollen‐disruption cassette driven by the *PG47* promoter, and an endosperm‐specific *DsRed* marker for seed sorting, but this system requires expensive optical sorters (Wu et al., 2016). Pioneer developed the *Ms44*‐based system (Fox et al., 2017). The Roundup® Hybridization System 2.0 induces male sterility through glyphosate‐activated RNAi, but its application requires precise herbicide dosage, timing, and strict field management (Yang et al., 2018). Most current technologies rely on exogenous visual or selectable markers and specialized sorting or management procedures, which raise biosafety concerns and limit large‐scale deployment. In addition, the widespread occurrence of heterofertilization remains a major bottleneck for achieving high seed purity in maize hybrid seed production.

To address these bottlenecks, we developed a novel smart double‐screening system termed the Seed and Seedling Marker Method (SSMM), which uses morphological markers derived from maize endogenous functional genes. The SSMM system integrates three functional components into a single construct: (i) The fertility restoration gene *Ms45*, which restores male fertility in male‐sterile lines; (ii) the *Mn1*/*Sh1‐*RNAi module driven by the *Mn1* native promoter, which generates a small‐kernel phenotype; and (iii) the visual marker gene *Lc* driven by the CaMV 35S promoter, providing a stable seedling color marker (Figure 1A).

**Figure 1:**
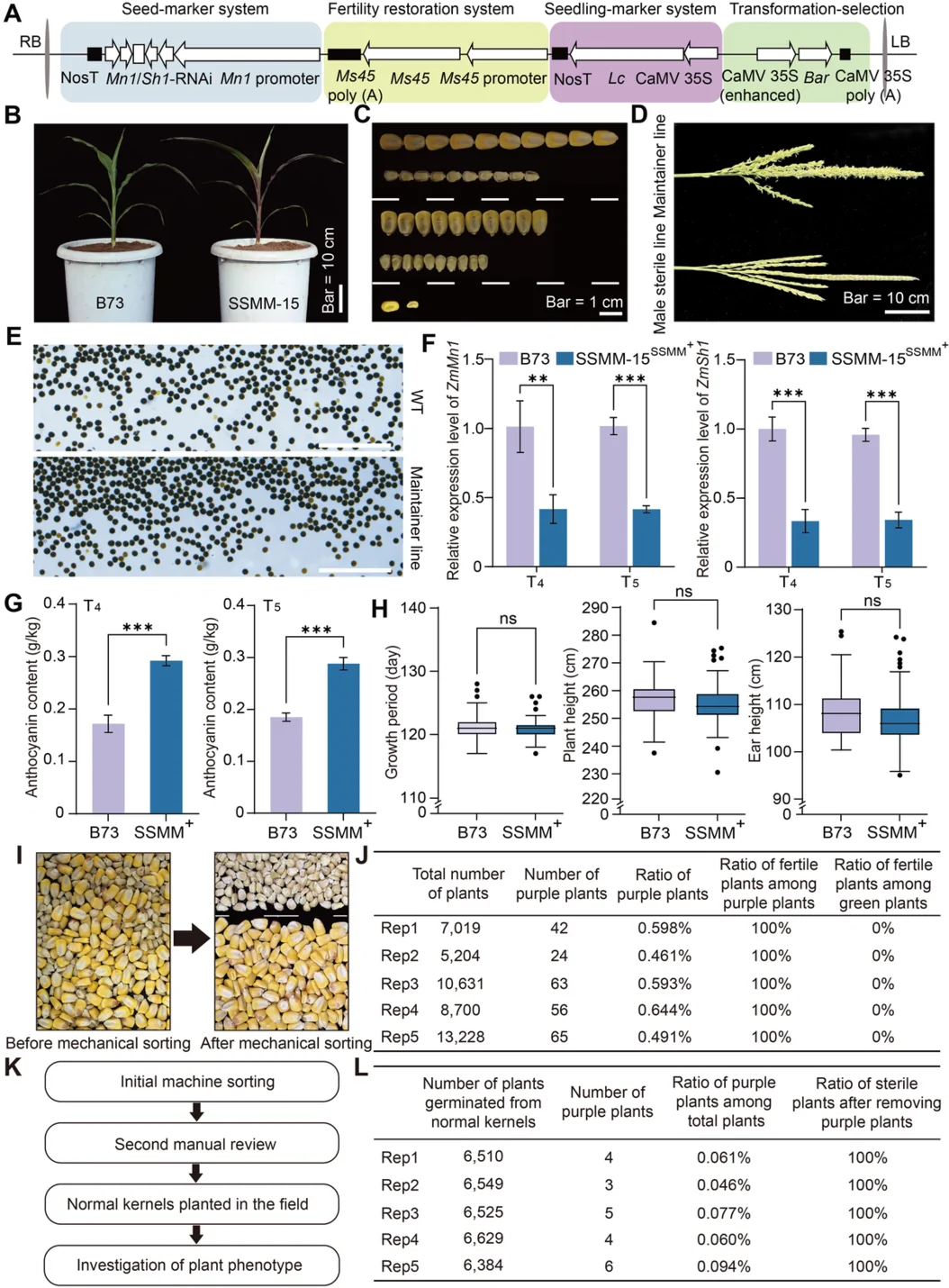
Overview and validation of the SSMM system for maize hybrid seed production **(A)** Schematic of the SSMM T‐DNA construct. **(B)** Phenotypic comparison of B73 and SSMM‐15 seedlings. Bar = 10 cm. **(C)** Kernel phenotypes of T_1_ ears produced by crosses between SSMM‐15 and the *ms45* mutant. Bar = 1 cm. **(D)** Tassels of maintainer line and male‐sterile line. Bar = 10 cm. **(E)** Microscopic views of I_2_‐KI‐stained pollen from B73 and the maintainer line. Bar = 1,000 μm. **(F)** Relative expression levels of *Mn1* and *Sh1* in 20‐DAP kernels (***P < 0.001). **(G)** Anthocyanin content in seedlings (***P < 0.001). **(H)** Agronomic trait comparison. ns, not significant. **(I)** High‐throughput mechanical sorting of male‐sterile kernels. **(J)** Frequency of fertile purple plants in field populations from mechanically sorted kernels. **(K)** Two‐step selection workflow for male‐sterile line propagation. **(L)** Efficacy of seedling color selection in eliminating heterofertilization‐derived escape plants.

The SSMM construct was introduced into the maize inbred line B73 via *Agrobacterium*‐mediated transformation, yielding 2,500 regenerated plants. Plants carrying the *Lc* gene exhibited distinct purple pigmentation, whereas non‐transgenic plants remained green, enabling rapid seedling‐stage discrimination. Subsequent herbicide‐resistance screening further validated positive transformants: All green plants failed to survive, whereas purple plants grew normally. Through this process, 20 T_0_ events with stable purple pigmentation were obtained. PCR analysis confirmed stable integration of *Ms45*, *Lc*, the *Mn1*/*Sh1*‐RNAi module, and *bar*. Each T_0_ event was crossed with the *ms45* mutant, and the resulting T_1_ ears showed a 1:1 segregation ratio of *SSMM* ^+^ to *SSMM* ^–^ kernels. Based on kernel size variation, five events exhibiting the most pronounced differences were selected for further multigenerational evaluation (T_2_–T_4_), among which SSMM‐15 showed the best overall performance and the most obvious kernel size segregation (Figure S1A). Further molecular characterization revealed that SSMM‐15 contained a single‐locus insertion comprising two tandemly arranged transgene copies, which segregated as a single genetic unit and was stably transmitted across generations (Figures S2, S3). In summary, we found that SSMM‐15 stably restored male fertility and stably displayed both small‐kernel and purple‐seedling phenotypes across generations, which established SSMM‐15 as a maintainer line for male‐sterile line propagation (Figures 1B–E, S4 and S5A).

To evaluate the reliability of SSMM‐15 during male‐sterile line propagation, we analyzed the activity of the *Mn1*/*Sh1*‐RNAi module and the expression of the *Lc* marker. In 20‐DAP *SSMM* ^+^ kernels from T_4_ and T_5_, 21–24 nt siRNAs were detected (Table S2). Compared with B73, *SSMM* ^+^ kernels exhibited significantly reduced *Mn1* and *Sh1* expression, demonstrating that this RNAi cassette exhibited stable and effective gene‐silencing activity across multiple generations (Figure 1F). Meanwhile, T_4_ and T_5_ *SSMM* ^+^ seedlings exhibited significantly higher *Lc* expression and a marked increase in leaf anthocyanin accumulation compared with non‐transgenic controls, suggesting that CaMV 35S‐driven *Lc* expression remained stable in advanced generations (Figures 1G, S5B). Together, these results indicate the effectiveness of the dual‐selection modules. The impact of the SSMM system on agronomic traits was further assessed. Except for reduced kernel size and purple plant coloration attributable to the SSMM cassette, no significant differences in other agronomic traits were observed compared with B73 (Figures 1H, S5C). Notably, *SSMM* ^+^ maintainer lines are used as pollen donors and are removed after pollination without harvesting kernels; therefore, these trait differences do not affect male‐sterile line propagation.

To meet commercial‐scale requirements, automated seed‐sorting machines were employed to evaluate high‐throughput selection based on kernel size. The purity of mechanically sorted male‐sterile seeds exceeded 99%. Errors arising from high‐throughput mechanical sorting based on kernel size can be effectively corrected at the seedling stage using plant color markers (Figure 1I, J; Table S3). To determine whether fertile purple plants detected in mechanically sorted male‐sterile populations were caused by sorting errors or heterofertilization, a second round of manual inspection was performed, followed by seedling‐stage phenotyping (Figure 1K). Among 6,510, 6,549, 6,525, 6,629, and 6,384 plants derived from normal‐sized kernels, 4, 3, 5, 4, and 6 purple plants were observed, respectively (Figure 1L). PCR analysis confirmed that all abnormal plants were transgenic *SSMM* ^+^ individuals, whereas their endosperm genotypes were consistent with the wild type (Figure S6). These results indicate that heterofertilization‐induced embryo‐endosperm genotype discordance prevents complete exclusion of fertile plants through kernel‐based selection alone. Because the *Lc*‐mediated seedling color marker reflects the embryo genotype, this second‐step selection enabled rapid identification and removal of these rare fertile escape plants. Fertility assessment at flowering confirmed that all remaining plants were male‐sterile (Figure 1L), demonstrating that the second‐step selection effectively compensates for heterofertilization‐associated limitations.

To assess the robustness of the SSMM system across diverse genetic backgrounds, the SSMM system was introgressed into five maize lines (LM4, X24621, JS005, Y98‐8, and JD60) through backcrossing (Figure S1B). In all backgrounds, crosses between maintainer and male‐sterile lines consistently produced a 1:1 segregation ratio of normal and small kernels, indicating stable kernel‐based selection (Figure S7; Table S1). *SSMM* ^+^ plants uniformly exhibited stable purple pigmentation at the seedling stage, whereas *SSMM* ^–^ plants maintained a normal green phenotype (Figure S7). Consistent with observations in the B73 background, no significant differences in agronomic traits were detected among *SSMM* ^+^ maintainer lines, male‐sterile lines, and their respective recurrent parents, except for kernel traits and plant coloration (Figure S8; Table S5). Field trials conducted in Sanya, Hainan and Zhangye, Gansu further demonstrated that the system exhibited stable kernel and seedling phenotypes across different ecological regions (Figure S7). To further evaluate the influence of genetic background, we incorporated the SSMM system into 329 maize inbred lines. Although the *SSMM* ^+^ small‐kernel phenotype was consistently distinguishable, visible anthocyanin pigmentation in *SSMM* ^+^ small kernels was observed in eight lines, representing 2.43% of the tested germplasm (Figure S9). This result suggests that kernel anthocyanin accumulation is highly dependent on genetic background, whereas the diagnostic small‐kernel phenotype and seedling coloration provide more reliable markers for selection. Collectively, these results demonstrate that the SSMM system is stably inherited across diverse maize germplasm and enables reliable two‐step selection largely independent of genetic background and environmental conditions, supporting its suitability for large‐scale hybrid seed production.

In summary, we developed a novel SSMM system to address the long‐standing challenges of low selection efficiency and the difficulty of ensuring seed purity in maize hybrid seed production. SSMM employs a two‐step marker design. The *Mn1*/*Sh1*‐RNAi seed marker enables high‐throughput enrichment of male‐sterile seeds using conventional seed sorters, which significantly reduces technical difficulty and screening costs. Meanwhile, the *Lc* visual seedling marker provides an embryo‐genotype‐dependent checkpoint that eliminates rare fertile escape plants and ensures high genetic purity. The system follows a “transgenic tools, non‐transgenic products” strategy, in which both the propagated male‐sterile lines and the resulting hybrids are free of transgenic elements. SSMM functions consistently across multiple generations and diverse genetic backgrounds, providing a practical platform for large‐scale hybrid seed production.

## CONFLICTS OF INTEREST

The authors declare no conflicts of interest.

## AUTHOR CONTRIBUTIONS

H.Z. conceived and designed the work. R.L. and S.H. jointly conducted the molecular experiments, collected and analyzed the data, wrote the manuscript, and prepared the figures. H.G. and Y.C. were responsible for vector construction. J.Y. oversaw field data collection and management. W.S., H.Z., Q.L., J.C., and J.L. contributed to manuscript revision. All authors read and approved the content.

## Supporting information

Additional Supporting Information may be found online in the supporting information tab for this article: http://onlinelibrary.wiley.com/doi/10.1111/jipb.70331/suppinfo

Figure S1. Schematic diagram of SSMM maintainer line development, screening, and production of diverse maintainer lines
Figure S2. Digital PCR‐based copy number determination
Figure S3. Detection of the tandem double‐copy structure
Figure S4. PCR confirmation of target elements across T_2_–T_4_ generations
Figure S5. Characterization of *SSMM* ^+^ plants
Figure S6. PCR analysis of abnormal purple plants
Figure S7. Robust performance of the SSMM system across diverse maize germplasm
Figure S8. Agronomic performance of diverse maize varieties introgressed with the SSMM system
Figure S9. Ear phenotypes showing anthocyanin pigmentation in kernels
Table S1. Segregation analysis of kernel size from crosses between *SSMM* ^+^ maintainer lines and *ms45* male‐sterile lines across five diverse maize backgrounds
Table S2. High‐throughput siRNA sequencing in multiple generations
Table S3. Analysis of mechanical sorting accuracy after introgression of the SSMM system into five maize inbred lines
Table S4. Primer information
Table S5. One‐way ANOVA results
