# Supplementary material for: Smart double‐screening system of propagating male‐sterile lines for maize hybrid seed production
Source: J Integr Plant Biol. 2026 Jun 23;68(11):3883–5. doi: 10.1111/jipb.70331 (PMC13725597; doi:10.1111/jipb.70331)
Supplement: Supplementary file 1 [file JIPB-68-3883-s001.docx]

**Supplemental Information**

**Figure S1. Schematic diagram of SSMM maintainer line development, screening, and production of diverse maintainer lines**

**Figure S2. Digital PCR-based copy number determination**

**Figure S3. Detection of the tandem double-copy structure**

**Figure S4. PCR confirmation of target elements across T_2_**–**T_4_ generations**

**Figure S5. Characterization of *SSMM*^+^ plants**

**Figure S6.** **PCR analysis of abnormal purple plants**

**Figure S7. Robust performance of the SSMM system across diverse maize germplasm**

**Figure S8. Agronomic performance of diverse maize varieties introgressed with the SSMM system**

**Figure S9. Ear phenotypes showing anthocyanin pigmentation in kernels**

**Table S1. Segregation analysis of kernel size from crosses between *SSMM*^+^ maintainer lines and *ms45* male-sterile lines across five diverse maize backgrounds**

**Table S2. High-throughput siRNA sequencing in multiple generations**

**Table S3. Analysis of mechanical sorting accuracy after introgression of the SSMM system into five maize inbred lines**

**Table S4. Primer information**

**Table S5. One-way ANOVA results**

**Figure S1. Schematic diagram of SSMM maintainer line development, screening, and production of diverse maintainer lines**

Schematic diagram of *Agrobacterium tumefaciens*-mediated transformation, seed- marker-based selection, seedling-marker-based selection, and molecular marker-assisted selection for generating elite SSMM transgenic maintainer lines.

(**A)** (i) *Agrobacterium*-mediated transformation and transgenic plant selection (using the *bar* gene and plant purple coloration as markers), followed by crossing with *ms45* mutants to generate T_1_ transgenic plants with *ms45* mutant site; (ii) Screening of transformants with significant variations in plant color and kernel size; (iii) Selection of transformants exhibiting fertility-restoration ability; (iv) Analysis of genetic stability among transformants, leading to the identification of the optimal transformant SSMM-15; (v) Development of the SSMM maintainer line and production of sterile lines.

**(B)** Production of diverse varieties of maintainer lines. Specifically, the SSMM maintainer is crossed with diverse maize varieties to introduce the SSMM; subsequent backcrossing and selection over multiple generations restores the recurrent parent's background, yielding a series of maintainer lines. Crossing these with their corresponding sterile lines enables the propagation of the male-sterile lines.

**Figure S2. Digital PCR-based copy number determination**

**(A–D)** Digital PCR amplification plots of *zSSIIb*, CaMV 35S, NOS, and *bar* genes, respectively. The upper dense cluster denotes positive droplets/microwells (containing target DNA with successful amplification and high fluorescence intensity), while the lower dense cluster denotes negative droplets/microwells (no target template or failed amplification with low fluorescence intensity). The horizontal line is the threshold (automatically set by the instrument/software) to discriminate positive and negative signals: droplets/microwells above the threshold are positive (target molecules present), and those below are negative (no target molecules).

**(E)** Because the tested plant material was heterozygous for the transgenic locus (*SSMM*^+^/−), whereas the endogenous reference gene *zSSIIb* is homozygous, the actual transgene copy number should be twice the measured value. Based on the pSSMM vector architecture, the *Ms45* gene is driven by its native promoter and terminator; the *Mn1*/*Sh1*-RNAi cassette is driven by the *Mn1* native promoter and terminated by the NOS terminator; the *Lc* gene is driven by the CaMV 35S promoter and terminated by the NOS terminator; and the bar selectable marker is driven by the CaMV 35S promoter and terminated by the CaMV terminator. Therefore, the pSSMM vector contains, in total, two CaMV 35S promoters, two NOS terminators, and one bar gene. Copy-number assays in the transformant SSMM-15 yielded measured copy numbers of four for the CaMV 35S promoters, four for the NOS terminator, and two for the bar gene. After correction for heterozygosity, SSMM-15 contains two pSSMM insertion copies in the recipient genome.

**Figure S3. Detection of the tandem double-copy structure**

The copy 1–copy 2 junction region was analyzed by Sanger sequencing, and the sequencing result was aligned with the reference sequence of the tandem double-copy vector. SnapGene visualization showed that the Sanger sequencing chromatogram matched the reference sequence across the junction region, confirming the correct linkage of the tandem double-copy structure. The arrow indicates the junction site between the end of copy 1 and the beginning of copy 2.

**Figure S4. PCR confirmation of target elements across T_2_**–**T_4_ generations**

H_2_O was used as the blank control; B73 genomic DNA as the negative control, and the SSMM vector DNA as the positive control to detect gene integration in materials from different generations. Primers used to amplify *Ms45*, the *Mn1*/*Sh1*-RNAi element, *Lc*, and bar were *Ms45*-f/*Ms45*-r, *Mn1*/*Sh1*-RNAi-f/*Mn1*/*Sh1*-RNAi-r, *Lc*-f/*Lc*-r, *bar*-f/*bar*-r (Table S4). -1, -2, and -3 represent three replicates. DNA Marker, DL2000.

**Figure S5. Characterization of *SSMM*^+^ plants  
Phenotypic analysis of** *SSMM*^−^ (normal) and *SSMM*^+^ (small) plants and kernels.

**(A)** Phenotypic comparison of *SSMM*^−^ (normal) and *SSMM*^+^ (small) kernels in segregating ears from progeny generated by crossing the SSMM-15 transformant with *ms45* sterile plants. (Tukey’s test; ***, *P* < 0.001).

**(B)** At the V7–V8 stage of the T_4_ and T_5_ generations, relative expression levels of the *Lc* in *SSMM*^+^ and WT leaves (Student’s *t*-test; ***, *P* < 0.001).

**(C)** Comparison of germination rate of *SSMM*^+^ and WT kernels (Student’s *t*-test; ns, not significant; *P* > 0.05). WT indicates B73.

**(D)** Comparison of 100-kernel weight between *SSMM*^−^ (normal) and *SSMM*^+^ (small) kernels from self-pollinated wild-type ears, self-pollinated ears of homozygous *SSMM*^+^ plants, and segregating ears derived from *SSMM*^+^ × *SSMM*^−^ crosses. WT indicates B73. (One-way ANOVA).

**(E)** Seedling vigor. Photographed at 20 days after emergence.

**Figure S6. PCR analysis of abnormal purple plants**

**(A)** Non-transgenic B73 (CK1) served as the negative control, and homozygous *SSMM^+^* plants (CK2) served as the positive control. Lanes 1–4, genomic DNA from four abnormal purple plants in the 6510 population; lanes 5–7, genomic DNA from three purple abnormal plants in the 6549 population; lanes 8–12, genomic DNA from five purple abnormal plants in the 6525 population; lanes 13–16, genomic DNA from four purple abnormal plants in the 6629 population; lanes 17–22, genomic DNA from six purple abnormal plants in the 6384 population. DNA Marker, DL2000.

**(B)** Endosperm genotyping of abnormal purple plants. Transgenic endosperm was used as the positive control (PC) and non-transgenic endosperm as the negative control (NC). Lanes 1–4, endosperm DNA from four abnormal samples in the 6510 population; lanes 5–7, endosperm DNA from three abnormal samples in the 6549 population; lanes 8–12, endosperm DNA from five abnormal samples in the 6525 population; lanes 13–16, endosperm DNA from four abnormal samples in the 6629 population; lanes 17–22, endosperm DNA from six abnormal samples in the 6384 population. All samples in lanes 1–22 showed the expected *zSSIIb* internal control band, whereas no transgene-specific amplification band was detected. DNA Marker, DL2000.

**Figure S7. Robust performance of the SSMM system across diverse maize germplasm**

Ear phenotypes of five diverse maize backgrounds (JS005, Y98-8, LM4, JD60, X24621) after introgression of the SSMM system into these backgrounds.

Seedling phenotypes of the five maize backgrounds in field plots. *SSMM*^+^ maintainer plants (purple seedlings); *SSMM*^–^ male-sterile plants (green seedlings).

**(A)** Sampling site: Sanya, Hainan, China (sown in September)

**(B)** Sampling site: Zhangye, Gansu, China (sown in April)

**Figure S8.** **Agronomic performance of diverse maize varieties introgressed with the SSMM system**

**(A–C)** Box plots showing no significant differences (ns, not significant; *P* > 0.05) in growth period **(A)**, plant height **(B)**, and ear height **(C)** among the recurrent parent (green), *SSMM*^–^ male-sterile line (purple), and *SSMM*^+^ maintainer line (blue) within each of five maize backgrounds (JS005, LM4, Y98-8, JD60, and X24621).

**Figure S9. Ear phenotypes showing anthocyanin pigmentation in kernels**

After the SSMM system was introduced into 329 maize inbred lines, purple kernels were observed in 8 of the 329 lines, accounting for 2.43%.

**(A)** After introduction of the SSMM system into one inbred line, all kernels of the transgenic maintainer line exhibited anthocyanin pigmentation.

**(B)** After introduction of the SSMM system into another inbred line, a subset of kernels from the transgenic maintainer line exhibited anthocyanin pigmentation during backcrossing. The triangles indicate *SSMM*^+^ kernels.

**Table S1. Segregation analysis of kernel size from crosses between *SSMM*^+^ maintainer lines and *ms45* male-sterile lines across five diverse maize backgrounds**

For each genetic background (JS005, LM4, Y98-8, JD60, and X24621), a total of 200 kernels from the middle segment of each ear were scored for normal kernels (*SSMM*^–^, male-sterile) and small kernels (*SSMM*^+^, maintainer). Chi-square (χ²) tests were performed to verify Mendelian segregation; all backgrounds exhibited a segregation ratio consistent with 1:1 (χ² < χ² _0.05, 1_ = 3.84, *P* > 0.05), confirming stable inheritance of the SSMM system and reliable kernel-based selection across diverse genetic backgrounds.

| Germplasm | Number of Samples | Normal Seeds | Small Seeds | χ² (1:1) | χ² _0.05,1_ (1:1) |
| --- | --- | --- | --- | --- | --- |
| JS005 | 200 | 109 | 91 | 1.445 | 3.84 |
|  | 200 | 92 | 108 | 1.125 |  |
|  | 200 | 97 | 103 | 0.125 |  |
|  | 200 | 113 | 87 | 3.125 |  |
|  | 200 | 100 | 100 | 0 |  |
|  | 200 | 113 | 87 | 3.125 |  |
|  | 200 | 104 | 96 | 0.245 |  |
|  | 200 | 98 | 102 | 0.045 |  |
|  | 200 | 106 | 94 | 0.605 |  |
|  | 200 | 101 | 99 | 0.005 |  |
| Total | 2000 | 1033 | 967 | 2.1125 |  |
| LM4 | 200 | 100 | 100 | 0 | 3.84 |
|  | 200 | 97 | 103 | 0.125 |  |
|  | 200 | 111 | 89 | 2.205 |  |
|  | 200 | 113 | 87 | 3.125 |  |
|  | 200 | 98 | 102 | 0.045 |  |
|  | 200 | 113 | 87 | 3.125 |  |
|  | 200 | 87 | 113 | 3.125 |  |
|  | 200 | 94 | 106 | 0.605 |  |
|  | 200 | 113 | 87 | 3.125 |  |
|  | 200 | 92 | 108 | 1.125 |  |
| Total | 2000 | 1018 | 982 | 0.648 |  |
| Y98-8 | 200 | 94 | 106 | 0.605 | 3.84 |
|  | 200 | 113 | 87 | 3.125 |  |
|  | 200 | 113 | 87 | 3.125 |  |
|  | 200 | 103 | 97 | 0.125 |  |
|  | 200 | 98 | 102 | 0.045 |  |
|  | 200 | 113 | 87 | 3.125 |  |
|  | 200 | 100 | 100 | 0 |  |
|  | 200 | 102 | 98 | 0.045 |  |
|  | 200 | 96 | 104 | 0.245 |  |
|  | 200 | 93 | 107 | 0.845 |  |
| Total | 2000 | 1025 | 975 | 1.2005 |  |
| JD60 | 200 | 102 | 98 | 0.045 | 3.84 |
|  | 200 | 102 | 98 | 0.045 |  |
|  | 200 | 102 | 98 | 0.045 |  |
|  | 200 | 90 | 110 | 1.805 |  |
|  | 200 | 102 | 98 | 0.045 |  |
|  | 200 | 99 | 101 | 0.005 |  |
|  | 200 | 100 | 100 | 0 |  |
|  | 200 | 99 | 101 | 0.005 |  |
|  | 200 | 95 | 105 | 0.405 |  |
|  | 200 | 110 | 90 | 1.805 |  |
| Total | 2000 | 1001 | 999 | 0.0005 |  |
| X24621 | 200 | 92 | 108 | 1.125 | 3.84 |
|  | 200 | 97 | 103 | 0.125 |  |
|  | 200 | 103 | 97 | 0.125 |  |
|  | 200 | 107 | 93 | 0.845 |  |
|  | 200 | 103 | 97 | 0.125 |  |
|  | 200 | 100 | 100 | 0 |  |
|  | 200 | 99 | 101 | 0.005 |  |
|  | 200 | 112 | 88 | 2.645 |  |
|  | 200 | 101 | 99 | 0.005 |  |
|  | 200 | 93 | 107 | 0.845 |  |
| Total | 2000 | 1007 | 993 | 0.0845 |  |

**Table S2. High-throughput siRNA sequencing in multiple generations**

High-throughput siRNA sequencing was performed on SSMM-15^SSMM+^ kernels at 20 DAP from the T_4_ and T_5_ generations.

| siRNA (21 nt) | | | Transcriptional direction | T_4_ generation read count | T_5_ generation read count |  |
| --- | --- | --- | --- | --- | --- | --- |
| TTCGGCGTGCTTGCTGCTGCT | | | + | 9 | 5 |  |
| TGCTGTTGCAGCTCGCAGGAG | | | – | 26 | 29 |  |
| TCGCAGGAGCGTCGCATGTCG | | | + | 38 | 33 |  |
| GCAGGAGCGTCGCATGTCGTC | | | – | 40 | 42 |  |
| CAGGAGCGTCGCATGTCGTCT | | | – | 41 | 37 |  |
| CAGGAGCGTCGCATGTCGTCT | | | + | 41 | 37 |  |
| AGGAGCGTCGCATGTCGTCTA | | | – | 42 | 48 |  |
| GGAGCGTCGCATGTCGTCTAC | | | – | 43 | 40 |  |
| GAGCGTCGCATGTCGTCTACA | | | – | 44 | 39 |  |
| AGCGTCGCATGTCGTCTACAA | | | – | 45 | 47 |  |
| TCGCATGTCGTCTACAACTAC | | | + | 49 | 233 |  |
| CGCATGTCGTCTACAACTACA | | | – | 50 | 52 |  |
| GCATGTCGTCTACAACTACAA | | | – | 51 | 47 |  |
| TGTCGTCTACAACTACAAGGA | | | – | 54 | 57 |  |
| CGTCTACAACTACAAGGACCT | | | – | 57 | 52 |  |
| GTCTACAACTACAAGGACCTC | | | – | 58 | 54 |  |
| CTACAACTACAAGGACCTCGA | | | – | 60 | 63 |  |
| TACAACTACAAGGACCTCGAA | | | – | 61 | 56 |  |
| ACAACTACAAGGACCTCGAAG | | | – | 62 | 64 |  |
| AACTACAAGGACCTCGAAGCC | | | + | 64 | 60 |  |
| ACTACAAGGACCTCGAAGCCG | | | – | 65 | 68 |  |
| CTACAAGGACCTCGAAGCCGA | | | – | 66 | 72 |  |
| CTCGAAGCCGAGGCTGCTGCG | | | + | 76 | 73 |  |
| CGAGGCTGCTGCGGCGACGGA | | | – | 84 | 79 |  |
| GGCTGCTGCGGCGACGGACCA | | | – | 87 | 89 |  |
| GCTGCTGCGGCGACGGACCAG | | | – | 88 | 234 |  |
| CTGCTGCGGCGACGGACCAGG | | | – | 89 | 91 |  |
| CAATGAACTGATAGCACTCTT | | | – | 432 | 427 |  |
| AATGAACTGATAGCACTCTTT | | | – | 433 | 429 |  |
| AATGAACTGATAGCACTCTTT | | | + | 433 | 429 |  |
|  | siRNA (21 nt) | | Transcriptional direction | T_4_ generation read count | T_5_ generation read count | |
|  | GGACCAGGTGCCGCCGTCCAT | | – | 102 | 98 | |
|  | ACCAGGTGCCGCCGTCCATCG | | – | 104 | 107 | |
|  | CCAGGTGCCGCCGTCCATCGT | | – | 105 | 100 | |
|  | CAGGTGCCGCCGTCCATCGTC | | + | 106 | 108 | |
|  | AGGTGCCGCCGTCCATCGTCA | | – | 107 | 103 | |
|  | AGGTGCCGCCGTCCATCGTCA | | + | 107 | 103 | |
|  | GGTGCCGCCGTCCATCGTCAA | | – | 108 | 114 | |
|  | GCCGCCGTCCATCGTCAACCC | | – | 111 | 108 | |
|  | GCCGTCCATCGTCAACCCCCT | | – | 114 | 109 | |
|  | CCGTCCATCGTCAACCCCCTG | | – | 115 | 117 | |
|  | CCGTCCATCGTCAACCCCCTG | | + | 115 | 117 | |
|  | GTCCATCGTCAACCCCCTGCT | | + | 117 | 119 | |
|  | TCCATCGTCAACCCCCTGCTC | | + | 118 | 114 | |
|  | CCATCGTCAACCCCCTGCTCA | | – | 119 | 122 | |
|  | ATCGTCAACCCCCTGCTCAGG | | + | 121 | 116 | |
|  | TCGTCAACCCCCTGCTCAGGA | | + | 122 | 118 | |
|  | TCAACCCCCTGCTCAGGACGG | | + | 125 | 128 | |
|  | CCTGCTCAGGACGGGGTACCA | | – | 132 | 127 | |
|  | TGCTCAGGACGGGGTACCACT | | + | 134 | 136 | |
|  | TGCTCAGGACGGGGTACCACT | | – | 134 | 136 | |
|  | GCTCAGGACGGGGTACCACTT | | – | 135 | 138 | |
|  | CTCAGGACGGGGTACCACTTC | | + | 136 | 142 | |
|  | TCAGGACGGGGTACCACTTCC | | + | 137 | 134 | |
|  | CAGGACGGGGTACCACTTCCA | | – | 138 | 133 | |
|  | AGGACGGGGTACCACTTCCAG | | + | 139 | 141 | |
|  | GGTACCACTTCCAGCCCCCCA | | – | 146 | 236 | |
|  | GTACCACTTCCAGCCCCCCAA | | – | 147 | 149 | |
|  | TACCACTTCCAGCCCCCCAAG | | + | 148 | 144 | |
|  | CAGCCCCCCAAGAACTGGATC | | + | 157 | 152 | |
|  | AGCCCCCCAAGAACTGGATCA | | – | 158 | 160 | |
|  | ATCAATGATCCCAACGCGCCC | | – | 175 | 171 | |
|  | TGCTGCGGCGACGGACCAGGT | | + | 90 | 86 | |
|  | TGCTGCGGCGACGGACCAGGT | | – | 90 | 86 | |
|  | ATCAATGATCCCAACGCGCCC | | + | 175 | 171 | |
|  | | siRNA (21 nt) | Transcriptional direction | T_4_ generation read count | T_5_ generation read count | |
|  | | TCAATGATCCCAACGCGCCCA | – | 176 | 182 | |
|  | | AATGATCCCAACGCGCCCATG | + | 178 | 175 | |
|  | | ATGATCCCAACGCGCCCATGT | + | 179 | 174 | |
|  | | TGATCCCAACGCGCCCATGTA | – | 180 | 182 | |
|  | | TGATCCCAACGCGCCCATGTA | + | 180 | 182 | |
|  | | TCCCAACGCGCCCATGTACTA | – | 183 | 185 | |
|  | | CCAACGCGCCCATGTACTACA | – | 185 | 181 | |
|  | | CAACGCGCCCATGTACTACAA | – | 186 | 189 | |
|  | | AACGCGCCCATGTACTACAAG | – | 187 | 182 | |
|  | | CATGTACTACAAGGGGTGGTA | – | 195 | 191 | |
|  | | ACTACAAGGGGTGGTACCATT | – | 200 | 203 | |
|  | | ACAAGGGGTGGTACCATTTCT | – | 203 | 198 | |
|  | | TACCATTTCTTCTACCAATAC | – | 214 | 216 | |
|  | | ACCATTTCTTCTACCAATACA | – | 215 | 211 | |
|  | | CCATTTCTTCTACCAATACAA | – | 216 | 219 | |
|  | | ATTTCTTCTACCAATACAATC | – | 218 | 224 | |
|  | | TCTTCTACCAATACAATCCCA | – | 221 | 218 | |
|  | | CTTCTACCAATACAATCCCAA | – | 222 | 217 | |
|  | | ACCAATACAATCCCAAGGGCG | – | 227 | 229 | |
|  | | CCAATACAATCCCAAGGGCGC | – | 228 | 238 | |
|  | | CAATACAATCCCAAGGGCGCC | + | 229 | 231 | |
|  | | AATACAATCCCAAGGGCGCCG | + | 230 | 226 | |
|  | | ATACAATCCCAAGGGCGCCGT | + | 231 | 234 | |
|  | | TACAATCCCAAGGGCGCCGTA | + | 232 | 227 | |
|  | | TACAATCCCAAGGGCGCCGTA | – | 232 | 227 | |
|  | | ACAATCCCAAGGGCGCCGTAT | – | 233 | 236 | |
|  | | CGTATGGGGCAACATCGTGTA | – | 249 | 255 | |
|  | | TATGGGGCAACATCGTGTATT | + | 251 | 248 | |
|  | | TATGGGGCAACATCGTGTATT | – | 251 | 248 | |
|  | | TGGGGCAACATCGTGTATTGG | – | 253 | 255 | |
|  | | TGGGGCAACATCGTGTATTGG | + | 253 | 255 | |
|  | | TGCGGCGACGGACCAGGTGCC | + | 93 | 88 | |
|  | | GCAACATCGTGTATTGGTCCC | – | 257 | 259 | |

|  | siRNA (21 nt) | Transcriptional direction | T_4_ generation read count | | T_5_ generation read count |  |
| --- | --- | --- | --- | --- | --- | --- |
|  | CAACATCGTGTATTGGTCCCT | – | 258 | | 254 |  |
|  | CAACATCGTGTATTGGTCCCT | + | 258 | | 254 |  |
|  | AACATCGTGTATTGGTCCCTC | + | 259 | | 254 |  |
|  | ACATCGTGTATTGGTCCCTCT | + | 260 | | 256 |  |
|  | ATCGTGTATTGGTCCCTCTCC | + | 262 | | 265 |  |
|  | TCGTGTATTGGTCCCTCTCCC | + | 263 | | 258 |  |
|  | TGTATTGGTCCCTCTCCCGTC | + | 266 | | 268 |  |
|  | TATTGGTCCCTCTCCCGTCCC | + | 268 | | 264 |  |
|  | ATTGGTCCCTCTCCCGTCCCA | + | 269 | | 272 |  |
|  | ATTGGTCCCTCTCCCGTCCCA | – | 269 | | 272 |  |
|  | TTGGTCCCTCTCCCGTCCCAG | + | 270 | | 267 |  |
|  | TGGTCCCTCTCCCGTCCCAGA | + | 271 | | 266 |  |
|  | TGGTCCCTCTCCCGTCCCAGA | – | 271 | | 266 |  |
|  | GTCCCTCTCCCGTCCCAGAGA | – | 273 | | 240 |  |
|  | TCCCTCTCCCGTCCCAGAGAA | + | 274 | | 276 |  |
|  | TCCCTCTCCCGTCCCAGAGAA | – | 274 | | 276 |  |
|  | CCCTCTCCCGTCCCAGAGAAA | – | 275 | | 278 |  |
|  | TCTCCCGTCCCAGAGAAACCC | + | 278 | | 273 |  |
|  | CTCCCGTCCCAGAGAAACCCT | + | 279 | | 275 |  |
|  | CTCCCGTCCCAGAGAAACCCT | – | 279 | | 275 |  |
|  | TCCCGTCCCAGAGAAACCCTC | + | 280 | | 275 |  |
|  | CGTCCCAGAGAAACCCTCCCT | – | 283 | | 285 |  |
|  | TCCCAGAGAAACCCTCCCTCC | + | 285 | | 281 |  |
|  | AAACCCTCCCTCCCTCCTCCA | + | 293 | | 295 |  |
|  | ACCCTCCCTCCCTCCTCCATT | + | 295 | | 241 |  |
|  | TCCCTCCCTCCTCCATTGGAC | + | 299 | | 301 |  |
|  | CCCTCCCTCCTCCATTGGACT | – | 300 | | 296 |  |
|  | CCCTCCCTCCTCCATTGGACT | + | 300 | | 296 |  |
|  | CCTCCCTCCTCCATTGGACTG | + | 301 | | 296 |  |
|  | CTCCCTCCTCCATTGGACTGC | + | 302 | | 298 |  |
|  | TCCCTCCTCCATTGGACTGCT | + | 303 | | 306 |  |
|  | TCCCTCCTCCATTGGACTGCT | – | 303 | | 306 |  |
|  | CCCTCCTCCATTGGACTGCTT | – | 304 | | 306 |  |
|  | CCTCCTCCATTGGACTGCTTG | + | 305 | | 301 |  |
| siRNA (21 nt) | | Transcriptional direction | | T_4_ generation read count | T_5_ generation read count | |
| TCCTCCATTGGACTGCTTGCT | | + | | 307 | 310 | |
| CTCCATTGGACTGCTTGCTCC | | + | | 309 | 315 | |
| TCCATTGGACTGCTTGCTCCC | | + | | 310 | 307 | |
| CCATTGGACTGCTTGCTCCCT | | + | | 311 | 306 | |
| ATTGGACTGCTTGCTCCCTGT | | + | | 313 | 315 | |
| TTGGACTGCTTGCTCCCTGTT | | + | | 314 | 242 | |
| TGGACTGCTTGCTCCCTGTTG | | + | | 315 | 317 | |
| GGACTGCTTGCTCCCTGTTGA | | – | | 316 | 312 | |
| CTGCTTGCTCCCTGTTGACCA | | – | | 319 | 322 | |
| TGCTTGCTCCCTGTTGACCAT | | – | | 320 | 315 | |
| TGCTTGCTCCCTGTTGACCAT | | + | | 320 | 315 | |
| GCTTGCTCCCTGTTGACCATT | | – | | 321 | 324 | |
| CTTGCTCCCTGTTGACCATTG | | + | | 322 | 317 | |
| TTGCTCCCTGTTGACCATTGG | | + | | 323 | 325 | |
| TTGACCATTGGGTATTCTGAA | | – | | 333 | 329 | |
| ACCATTGGGTATTCTGAACCA | | – | | 336 | 339 | |
| ACCATTGGGTATTCTGAACCA | | + | | 336 | 339 | |
| CATTGGGTATTCTGAACCATC | | + | | 338 | 335 | |
| TTGGGTATTCTGAACCATCGA | | + | | 340 | 335 | |
| ATTCTGAACCATCGAGCCATG | | + | | 346 | 349 | |
| ATTCTGAACCATCGAGCCATG | | – | | 346 | 349 | |
| TTCTGAACCATCGAGCCATGG | | – | | 347 | 343 | |
| TCTGAACCATCGAGCCATGGC | | + | | 348 | 351 | |
| AACCATCGAGCCATGGCTGCC | | + | | 352 | 347 | |
| ACCATCGAGCCATGGCTGCCA | | – | | 353 | 355 | |
| CCATCGAGCCATGGCTGCCAA | | – | | 354 | 350 | |
| CATCGAGCCATGGCTGCCAAG | | + | | 355 | 358 | |
| ATCGAGCCATGGCTGCCAAGC | | + | | 356 | 362 | |
| TCGAGCCATGGCTGCCAAGCT | | + | | 357 | 354 | |
| TCGAGCCATGGCTGCCAAGCT | | – | | 357 | 354 | |
| CGAGCCATGGCTGCCAAGCTG | | + | | 358 | 360 | |
| GAGCCATGGCTGCCAAGCTGA | | – | | 359 | 244 | |
| GCCATGGCTGCCAAGCTGACT | | – | | 361 | 363 | |
| ATGGCTGCCAAGCTGACTCGC | | + | | 364 | 360 | |

| siRNA (21 nt) | Transcriptional direction | T_4_ generation read count | T_5_ generation read count |  |  |
| --- | --- | --- | --- | --- | --- |
| TGGTGCCACCTTCTCCTCCCA | + | 408 | 411 |  |  |
| TGGTGCCACCTTCTCCTCCCA | – | 408 | 411 |  |  |
| TGCCACCTTCTCCTCCCATCC | + | 411 | 413 |  |  |
| CCACCTTCTCCTCCCATCCCA | + | 413 | 409 |  |  |
| TGGCTGCCAAGCTGACTCGCC | + | 365 | 368 |  |  |
| GCTGCCAAGCTGACTCGCCTC | – | 367 | 362 |  |  |
| CTGCCAAGCTGACTCGCCTCC | + | 368 | 364 |  |  |
| TGCCAAGCTGACTCGCCTCCA | + | 369 | 372 |  |  |
| CCAAGCTGACTCGCCTCCACA | – | 371 | 366 |  |  |
| CAAGCTGACTCGCCTCCACAG | – | 372 | 374 |  |  |
| GCTGACTCGCCTCCACAGTCT | – | 375 | 371 |  |  |
| CTGACTCGCCTCCACAGTCTT | – | 376 | 379 |  |  |
| CTCGCCTCCACAGTCTTCGCG | + | 380 | 386 |  |  |
| TCGCCTCCACAGTCTTCGCGA | – | 381 | 378 |  |  |
| CGCCTCCACAGTCTTCGCGAA | – | 382 | 377 |  |  |
| GCCTCCACAGTCTTCGCGAAC | – | 383 | 385 |  |  |
| CCTCCACAGTCTTCGCGAACG | – | 384 | 386 |  |  |
| CCTCCACAGTCTTCGCGAACG | + | 384 | 386 |  |  |
| TCCACAGTCTTCGCGAACGCC | + | 386 | 382 |  |  |
| AGTCTTCGCGAACGCCTTGGT | + | 391 | 386 |  |  |
| GTCTTCGCGAACGCCTTGGTG | + | 392 | 394 |  |  |
| TCTTCGCGAACGCCTTGGTGC | + | 393 | 389 |  |  |
| CTTCGCGAACGCCTTGGTGCC | + | 394 | 397 |  |  |
| TTCGCGAACGCCTTGGTGCCA | – | 395 | 401 |  |  |
| TTCGCGAACGCCTTGGTGCCA | + | 395 | 401 |  |  |
| TCGCGAACGCCTTGGTGCCAC | + | 396 | 391 |  |  |
| CGCGAACGCCTTGGTGCCACC | + | 397 | 399 |  |  |
| GCGAACGCCTTGGTGCCACCT | – | 398 | 246 |  |  |
| CGAACGCCTTGGTGCCACCTT | – | 399 | 401 |  |  |
| AACGCCTTGGTGCCACCTTCT | + | 401 | 397 |  |  |
| AACGCCTTGGTGCCACCTTCT | – | 401 | 397 |  |  |
| GCCTTGGTGCCACCTTCTCCT | – | 404 | 399 |  |  |
| TTGGTGCCACCTTCTCCTCCC | + | 407 | 403 |  |  |
| CACCTTCTCCTCCCATCCCAA | – | 414 | 417 |  |  |
| siRNA (21 nt) | Transcriptional direction | T_4_ generation read count | T_5_ generation read count | |  |
| TTCACCAGGGCAAGGGAATGC | + | 464 | 459 | |  |
| TCACCAGGGCAAGGGAATGCT | + | 465 | 467 | |  |
| TCACCAGGGCAAGGGAATGCT | – | 465 | 467 | |  |
| CACCAGGGCAAGGGAATGCTT | – | 466 | 468 | |  |
| ACCAGGGCAAGGGAATGCTTC | + | 467 | 463 | |  |
| ACCTTCTCCTCCCATCCCAAT | – | 415 | 421 | |  |
| CTTCTCCTCCCATCCCAATGA | – | 417 | 414 | |  |
| TTCTCCTCCCATCCCAATGAA | – | 418 | 413 | |  |
| TTCTCCTCCCATCCCAATGAA | + | 418 | 413 | |  |
| TCTCCTCCCATCCCAATGAAC | – | 419 | 421 | |  |
| TCTCCTCCCATCCCAATGAAC | + | 419 | 421 | |  |
| CTCCTCCCATCCCAATGAACT | – | 420 | 416 | |  |
| TCCTCCCATCCCAATGAACTG | + | 421 | 424 | |  |
| CTCCCATCCCAATGAACTGAT | – | 423 | 418 | |  |
| TCCCATCCCAATGAACTGATA | – | 424 | 420 | |  |
| TCCCAATGAACTGATAGCACT | – | 429 | 432 | |  |
| ATGAACTGATAGCACTCTTTT | + | 434 | 440 | |  |
| TGAACTGATAGCACTCTTTTC | + | 435 | 432 | |  |
| AACTGATAGCACTCTTTTCCA | + | 437 | 432 | |  |
| AACTGATAGCACTCTTTTCCA | – | 437 | 432 | |  |
| ACTGATAGCACTCTTTTCCAG | + | 438 | 248 | |  |
| CTGATAGCACTCTTTTCCAGG | + | 439 | 441 | |  |
| TGATAGCACTCTTTTCCAGGT | + | 440 | 436 | |  |
| GATAGCACTCTTTTCCAGGTA | + | 441 | 444 | |  |
| GATAGCACTCTTTTCCAGGTA | – | 441 | 444 | |  |
| ATAGCACTCTTTTCCAGGTAT | + | 442 | 438 | |  |
| TAGCACTCTTTTCCAGGTATG | + | 443 | 446 | |  |
| TCTTTTCCAGGTATGTTCACC | + | 449 | 444 | |  |
| TTTTCCAGGTATGTTCACCAG | + | 451 | 453 | |  |
| TTTCCAGGTATGTTCACCAGG | + | 452 | 448 | |  |
| TATGTTCACCAGGGCAAGGGA | – | 460 | 463 | |  |
| ATGTTCACCAGGGCAAGGGAA | – | 461 | 467 | |  |
| TGTTCACCAGGGCAAGGGAAT | – | 462 | 459 | |  |
| CCAGGGCAAGGGAATGCTTCA | – | 468 | 471 | |  |
| siRNA (21 nt) | Transcriptional direction | T_4_ generation read count | T_5_ generation read count | | |
| ACCACTTCCAGCCCCCCAAGA | – | 149 | 152 | | |
| CCACTTCCAGCCCCCCAAGAA | – | 150 | 145 | | |
| ACTTCCAGCCCCCCAAGAACT | – | 152 | 148 | | |
| CTTCCAGCCCCCCAAGAACTG | + | 153 | 156 | | |
| AATCCCAAGGGCGCCGTATGG | + | 235 | 230 | | |
| CCAAGGGCGCCGTATGGGGCA | – | 239 | 241 | | |
| CAAGGGCGCCGTATGGGGCAA | – | 240 | 236 | | |
| GGGCGCCGTATGGGGCAACAT | – | 243 | 246 | | |
| CCAGAGAAACCCTCCCTCCCT | – | 287 | 290 | | |
| CAGAGAAACCCTCCCTCCCTC | + | 288 | 294 | | |
| AGAGAAACCCTCCCTCCCTCC | + | 289 | 286 | | |
| AGAAACCCTCCCTCCCTCCTC | + | 291 | 286 | | |
| TGGGTATTCTGAACCATCGAG | – | 341 | 343 | | |
| GTATTCTGAACCATCGAGCCA | – | 344 | 243 | | |
| TATTCTGAACCATCGAGCCAT | – | 345 | 347 | | |
| TATTCTGAACCATCGAGCCAT | + | 345 | 347 | | |
| CACAGTCTTCGCGAACGCCTT | + | 388 | 391 | | |
| CACAGTCTTCGCGAACGCCTT | – | 388 | 391 | | |
| ACAGTCTTCGCGAACGCCTTG | + | 389 | 385 | | |
| CAGTCTTCGCGAACGCCTTGG | + | 390 | 393 | | |
| CAATGAACTGATAGCACTCTT | + | 432 | 427 | | |

| siRNA (22 nt) | Transcriptional direction | T_4_ generation read count | T_5_ generation read count |
| --- | --- | --- | --- |
| CTACAACTACAAGGACCTCGAA | – | 60 | 63 |
| AGGCTGCTGCGGCGACGGACCA | + | 86 | 81 |
| GGACCAGGTGCCGCCGTCCATC | – | 102 | 97 |
| ACCAGGTGCCGCCGTCCATCGT | – | 104 | 100 |
| ATCGTCAACCCCCTGCTCAGGA | + | 121 | 116 |
| AGGTGCCGCCGTCCATCGTCAA | + | 107 | 103 |
| TGCCGCCGTCCATCGTCAACCC | + | 110 | 116 |
| CCCTCCTCCATTGGACTGCTTG | + | 304 | 306 |
| CCTCCATTGGACTGCTTGCTCC | + | 308 | 304 |
| TCCATTGGACTGCTTGCTCCCT | + | 310 | 313 |

| siRNA (22 nt) | | Transcriptional direction | T_4_ generation read count | T_5_ generation read count | |  |
| --- | --- | --- | --- | --- | --- | --- |
| TTGGACTGCTTGCTCCCTGTTG | | + | 314 | 317 | |  |
| TGCTTGCTCCCTGTTGACCATT | | + | 320 | 315 | |  |
| CTTGCTCCCTGTTGACCATTGG | | + | 322 | 317 | |  |
| TGACCATTGGGTATTCTGAACC | | + | 334 | 330 | |  |
| GGTATTCTGAACCATCGAGCCA | | – | 343 | 339 | |  |
| TTCTGAACCATCGAGCCATGGC | | + | 347 | 353 | |  |
| AACCATCGAGCCATGGCTGCCA | | + | 352 | 349 | |  |
| ACCATCGAGCCATGGCTGCCAA | | – | 353 | 348 | |  |
| CCATCGAGCCATGGCTGCCAAG | | + | 354 | 349 | |  |
| TCGAGCCATGGCTGCCAAGCTG | | + | 357 | 251 | |  |
| CGAGCCATGGCTGCCAAGCTGA | | + | 358 | 360 | |  |
| GAGCCATGGCTGCCAAGCTGAC | | + | 359 | 355 | |  |
| CCATGGCTGCCAAGCTGACTCG | | – | 362 | 365 | |  |
| ATGGCTGCCAAGCTGACTCGCC | | + | 364 | 367 | |  |
| GCTGCCAAGCTGACTCGCCTCC | | + | 367 | 370 | |  |
| GCTGACTCGCCTCCACAGTCTT | | – | 375 | 370 | |  |
| CTCGCCTCCACAGTCTTCGCGA | | – | 380 | 375 | |  |
| TCGCCTCCACAGTCTTCGCGAA | | – | 381 | 377 | |  |
| CGCCTCCACAGTCTTCGCGAAC | | – | 382 | 378 | |  |
| TCCACAGTCTTCGCGAACGCCT | | + | 386 | 392 | |  |
| CCACAGTCTTCGCGAACGCCTT | | – | 387 | 384 | |  |
| CACAGTCTTCGCGAACGCCTTG | | – | 388 | 383 | |  |
| ACAGTCTTCGCGAACGCCTTGG | | + | 389 | 384 | |  |
| CAGTCTTCGCGAACGCCTTGGT | | + | 390 | 252 | |  |
| TCTTCGCGAACGCCTTGGTGCC | | + | 393 | 395 | |  |
| CTTCGCGAACGCCTTGGTGCCA | | – | 394 | 390 | |  |
| TTCGCGAACGCCTTGGTGCCAC | | + | 395 | 398 | |  |
| TCGCGAACGCCTTGGTGCCACC | | + | 396 | 399 | |  |
| CGCGAACGCCTTGGTGCCACCT | | + | 397 | 400 | |  |
| GCGAACGCCTTGGTGCCACCTT | | + | 398 | 393 | |  |
| CCTTGGTGCCACCTTCTCCTCC | | + | 405 | 400 | |  |
| CTTGGTGCCACCTTCTCCTCCC | | + | 406 | 402 | |  |
| TTGGTGCCACCTTCTCCTCCCA | | + | 407 | 403 | |  |
| CCGCCGTCCATCGTCAACCCCC | | + | 112 | 109 | |  |
|  | siRNA (22 nt) | Transcriptional direction | T_4_ generation read count | | T_5_ generation read count | |
|  | GCCACCTTCTCCTCCCATCCCA | + | 412 | | 418 | |
|  | CCACCTTCTCCTCCCATCCCAA | + | 413 | | 410 | |
|  | CCACCTTCTCCTCCCATCCCAA | – | 413 | | 410 | |
|  | ACCTTCTCCTCCCATCCCAATG | – | 415 | | 410 | |
|  | CTTCTCCTCCCATCCCAATGAA | – | 417 | | 253 | |
|  | TTCTCCTCCCATCCCAATGAAC | + | 418 | | 420 | |
|  | CTCCTCCCATCCCAATGAACTG | + | 420 | | 416 | |
|  | CTCCTCCCATCCCAATGAACTG | – | 420 | | 416 | |
|  | CCTCCCATCCCAATGAACTGAT | – | 422 | | 425 | |
|  | TCCCATCCCAATGAACTGATAG | + | 424 | | 427 | |
|  | ATGAACTGATAGCACTCTTTTC | + | 434 | | 429 | |
|  | GAACTGATAGCACTCTTTTCCA | – | 436 | | 431 | |
|  | AACTGATAGCACTCTTTTCCAG | + | 437 | | 433 | |
|  | ACTGATAGCACTCTTTTCCAGG | + | 438 | | 434 | |
|  | CTGATAGCACTCTTTTCCAGGT | + | 439 | | 445 | |
|  | TGATAGCACTCTTTTCCAGGTA | + | 440 | | 437 | |
|  | GATAGCACTCTTTTCCAGGTAT | – | 441 | | 436 | |
|  | ATAGCACTCTTTTCCAGGTATG | + | 442 | | 437 | |
|  | TTTTCCAGGTATGTTCACCAGG | + | 451 | | 254 | |
|  | TATGTTCACCAGGGCAAGGGAA | – | 460 | | 462 | |
|  | ATGTTCACCAGGGCAAGGGAAT | – | 461 | | 457 | |
|  | CACCAGGGCAAGGGAATGCTTC | + | 466 | | 469 | |
|  | CACCAGGGCAAGGGAATGCTTC | – | 466 | | 469 | |
|  | ACCAGGGCAAGGGAATGCTTCA | + | 467 | | 470 | |
|  | CCTGCTCAGGACGGGGTACCAC | – | 132 | | 127 | |
|  | TGCTCAGGACGGGGTACCACTT | + | 134 | | 136 | |
|  | TGCTCAGGACGGGGTACCACTT | – | 134 | | 136 | |
|  | CTCAGGACGGGGTACCACTTCC | + | 136 | | 132 | |
|  | ATGATCCCAACGCGCCCATGTA | + | 179 | | 182 | |
|  | TACTACAAGGGGTGGTACCATT | – | 199 | | 202 | |
|  | ACCAATACAATCCCAAGGGCGC | – | 227 | | 230 | |
|  | AATCCCAAGGGCGCCGTATGGG | + | 235 | | 241 | |
|  | AATACAATCCCAAGGGCGCCGT | – | 230 | | 225 | |
|  | AATACAATCCCAAGGGCGCCGT | + | 230 | | 225 | |

| siRNA (22 nt) | Transcriptional direction | T_4_ generation read count | T_5_ generation read count |
| --- | --- | --- | --- |
| ATACAATCCCAAGGGCGCCGTA | + | 231 | 227 |
| ATGGGGCAACATCGTGTATTGG | + | 252 | 249 |
| AACATCGTGTATTGGTCCCTCT | + | 259 | 254 |
| TGTATTGGTCCCTCTCCCGTCC | + | 266 | 261 |
| TATTGGTCCCTCTCCCGTCCCA | + | 268 | 249 |
| ATTGGTCCCTCTCCCGTCCCAG | + | 269 | 271 |
| TTGGTCCCTCTCCCGTCCCAGA | + | 270 | 266 |
| TCTCCCGTCCCAGAGAAACCCT | + | 278 | 281 |
| TCTCCCGTCCCAGAGAAACCCT | – | 278 | 281 |
| CTCCCGTCCCAGAGAAACCCTC | + | 279 | 282 |
| TCCCGTCCCAGAGAAACCCTCC | + | 280 | 275 |
| CCGTCCCAGAGAAACCCTCCCT | + | 282 | 277 |
| CGTCCCAGAGAAACCCTCCCTC | + | 283 | 279 |
| TCCCAGAGAAACCCTCCCTCCC | + | 285 | 281 |
| AACCCTCCCTCCCTCCTCCATT | + | 294 | 300 |
| CTCCCTCCCTCCTCCATTGGAC | + | 298 | 295 |
| CCTCCCTCCTCCATTGGACTGC | + | 301 | 296 |
| TCCCTCCTCCATTGGACTGCTT | + | 303 | 298 |
| TCCCTCCTCCATTGGACTGCTT | – | 303 | 298 |
| ATTGGACTGCTTGCTCCCTGTT | + | 313 | 316 |

| siRNA (23 nt) | Transcriptional direction | T_4_ generation read count | T_5_ generation read count |
| --- | --- | --- | --- |
| ATCAATGATCCCAACGCGCCCAT | + | 175 | 178 |
| ATCCCAACGCGCCCATGTACTAC | + | 182 | 185 |
| ACCATTTCTTCTACCAATACAAT | – | 215 | 218 |
| CAATACAATCCCAAGGGCGCCGT | – | 229 | 224 |
| AATACAATCCCAAGGGCGCCGTA | – | 230 | 225 |
| AGGTATGTTCACCAGGGCAAGGG | + | 457 | 453 |
| GGTATGTTCACCAGGGCAAGGGA | + | 458 | 454 |
| GTATGTTCACCAGGGCAAGGGAA | – | 459 | 465 |
| TGTTCACCAGGGCAAGGGAATGC | + | 462 | 457 |
| GTTCACCAGGGCAAGGGAATGCT | – | 463 | 458 |
| CACCAGGGCAAGGGAATGCTTCA | – | 466 | 461 |

| siRNA (23 nt) | Transcriptional direction | T_4_ generation read count | T_5_ generation read count |
| --- | --- | --- | --- |
| ACATCGTGTATTGGTCCCTCTCC | + | 260 | 256 |
| GTCCCTCTCCCGTCCCAGAGAAA | – | 273 | 269 |
| ATTGGACTGCTTGCTCCCTGTTG | + | 313 | 319 |
| TTGGACTGCTTGCTCCCTGTTGA | + | 314 | 311 |
| GCTCCCTGTTGACCATTGGGTAT | – | 325 | 320 |
| GGTATTCTGAACCATCGAGCCAT | – | 343 | 338 |
| ATTCTGAACCATCGAGCCATGGC | + | 346 | 341 |
| ATCGAGCCATGGCTGCCAAGCTG | + | 356 | 358 |
| TCGAGCCATGGCTGCCAAGCTGA | + | 357 | 359 |
| TGGCTGCCAAGCTGACTCGCCTC | + | 365 | 361 |
| AGCTGACTCGCCTCCACAGTCTT | – | 374 | 377 |
| ACTCGCCTCCACAGTCTTCGCGA | – | 379 | 382 |
| CTCGCCTCCACAGTCTTCGCGAA | – | 380 | 383 |
| TCGCCTCCACAGTCTTCGCGAAC | + | 381 | 376 |
| CGCCTCCACAGTCTTCGCGAACG | – | 382 | 377 |
| ACAGTCTTCGCGAACGCCTTGGT | + | 389 | 385 |
| AGTCTTCGCGAACGCCTTGGTGC | + | 391 | 387 |
| TTCGCGAACGCCTTGGTGCCACC | + | 395 | 401 |
| CCTTGGTGCCACCTTCTCCTCCC | + | 405 | 402 |
| GTGCCACCTTCTCCTCCCATCCC | + | 410 | 405 |
| ACCTTCTCCTCCCATCCCAATGA | + | 415 | 410 |
| TCCTCCCATCCCAATGAACTGAT | – | 421 | 416 |
| TCCCAATGAACTGATAGCACTCT | – | 429 | 431 |
| CCCAATGAACTGATAGCACTCTT | – | 430 | 432 |
| ATGAACTGATAGCACTCTTTTCC | + | 434 | 430 |
| GAACTGATAGCACTCTTTTCCAG | + | 436 | 439 |
| AACTGATAGCACTCTTTTCCAGG | + | 437 | 440 |
| ACTGATAGCACTCTTTTCCAGGT | + | 438 | 441 |
| CTGATAGCACTCTTTTCCAGGTA | + | 439 | 434 |
| CTTTTCCAGGTATGTTCACCAGG | + | 450 | 445 |
| CGCAGGAGCGTCGCATGTCGTCT | – | 39 | 41 |
| AGCCGAGGCTGCTGCGGCGACGG | + | 81 | 83 |
| CCTGCTCAGGACGGGGTACCACT | – | 132 | 128 |
| TATGTTCACCAGGGCAAGGGAAT | – | 460 | 457 |

| siRNA (24 nt) | | | Transcriptional direction | T_4_ generation read count | T_5_ generation read count |  |
| --- | --- | --- | --- | --- | --- | --- |
| TGTCGTCTACAACTACAAGGACCT | | | – | 54 | 49 |  |
| GTCTACAACTACAAGGACCTCGAA | | | – | 58 | 60 |  |
| AAGCCGAGGCTGCTGCGGCGACGG | | | + | 80 | 76 |  |
| GGCTGCTGCGGCGACGGACCAGGT | | | – | 87 | 90 |  |
| GGACCAGGTGCCGCCGTCCATCGT | | | – | 102 | 108 |  |
| GCCGCCGTCCATCGTCAACCCCCT | | | – | 111 | 108 |  |
| GCCGTCCATCGTCAACCCCCTGCT | | | – | 114 | 109 |  |
| TCCATCGTCAACCCCCTGCTCAGG | | | + | 118 | 120 |  |
| ATCGTCAACCCCCTGCTCAGGACG | | | + | 121 | 117 |  |
| CCCTGCTCAGGACGGGGTACCACT | | | – | 131 | 134 |  |
| CCTGCTCAGGACGGGGTACCACTT | | | – | 132 | 127 |  |
| AGGACGGGGTACCACTTCCAGCCC | | | + | 139 | 141 |  |
| TACCATTTCTTCTACCAATACAAT | | | – | 214 | 209 |  |
| CATTTCTTCTACCAATACAATCCC | | | – | 217 | 219 |  |
| CCAATACAATCCCAAGGGCGCCGT | | | – | 228 | 224 |  |
| AATACAATCCCAAGGGCGCCGTAT | | | – | 230 | 233 |  |
| ATACAATCCCAAGGGCGCCGTATG | | | + | 231 | 237 |  |
| AATCCCAAGGGCGCCGTATGGGGC | | | + | 235 | 232 |  |
| CAAGGGCGCCGTATGGGGCAACAT | | | – | 240 | 235 |  |
| AAGGGCGCCGTATGGGGCAACATC | | | + | 241 | 243 |  |
| GCCGTATGGGGCAACATCGTGTAT | | | – | 247 | 243 |  |
| AACATCGTGTATTGGTCCCTCTCC | | | + | 259 | 262 |  |
| ACATCGTGTATTGGTCCCTCTCCC | | | + | 260 | 255 |  |
| ATTGGTCCCTCTCCCGTCCCAGAG | | | + | 269 | 271 |  |
| CCTCTCCCGTCCCAGAGAAACCCT | | | – | 276 | 272 |  |
| TCTCCCGTCCCAGAGAAACCCTCC | | | + | 278 | 281 |  |
| TCCCGTCCCAGAGAAACCCTCCCT | | | + | 280 | 286 |  |
| CCGTCCCAGAGAAACCCTCCCTCC | | | – | 282 | 279 |  |
| CACTCTTTTCCAGGTATGTTCACC | | | + | 446 | 442 |  |
| CAGGTATGTTCACCAGGGCAAGGG | | | + | 456 | 459 |  |
| AGGTATGTTCACCAGGGCAAGGGA | | | + | 457 | 452 |  |
| GGTATGTTCACCAGGGCAAGGGAA | | | – | 458 | 460 |  |
| GAACTGATAGCACTCTTTTCCAGG | | | + | 436 | 433 |  |
| AACTGATAGCACTCTTTTCCAGGT | | | + | 437 | 432 |  |
|  | siRNA (24 nt) | Transcriptional direction | | T_4_ generation read count | T_5_ generation read count |  |
|  | ATTCTGAACCATCGAGCCATGGCT | + | | 346 | 342 |  |
|  | AACCATCGAGCCATGGCTGCCAAG | + | | 352 | 355 |  |
|  | ACCATCGAGCCATGGCTGCCAAGC | + | | 353 | 348 |  |
|  | CATCGAGCCATGGCTGCCAAGCTG | + | | 355 | 357 |  |
|  | ATCGAGCCATGGCTGCCAAGCTGA | + | | 356 | 352 |  |
|  | TCGAGCCATGGCTGCCAAGCTGAC | + | | 357 | 360 |  |
|  | CGAGCCATGGCTGCCAAGCTGACT | – | | 358 | 364 |  |
|  | CGAGCCATGGCTGCCAAGCTGACT | + | | 358 | 364 |  |
|  | AGCCATGGCTGCCAAGCTGACTCG | + | | 360 | 355 |  |
|  | CCATGGCTGCCAAGCTGACTCGCC | + | | 362 | 364 |  |
|  | CATGGCTGCCAAGCTGACTCGCCT | + | | 363 | 359 |  |
|  | AGAGAAACCCTCCCTCCCTCCTCC | + | | 289 | 284 |  |
|  | AAACCCTCCCTCCCTCCTCCATTG | + | | 293 | 295 |  |
|  | AACCCTCCCTCCCTCCTCCATTGG | + | | 294 | 290 |  |
|  | ACCCTCCCTCCCTCCTCCATTGGA | + | | 295 | 298 |  |
|  | CTCCCTCCCTCCTCCATTGGACTG | + | | 298 | 293 |  |
|  | CCCTCCCTCCTCCATTGGACTGCT | – | | 300 | 302 |  |
|  | CCCTCCCTCCTCCATTGGACTGCT | + | | 300 | 302 |  |
|  | CCTCCCTCCTCCATTGGACTGCTT | – | | 301 | 304 |  |
|  | CTCCCTCCTCCATTGGACTGCTTG | + | | 302 | 308 |  |
|  | TCCTCCATTGGACTGCTTGCTCCC | + | | 307 | 304 |  |
|  | ATTGGACTGCTTGCTCCCTGTTGA | + | | 313 | 308 |  |
|  | TTGGACTGCTTGCTCCCTGTTGAC | + | | 314 | 316 |  |
|  | TGGACTGCTTGCTCCCTGTTGACC | + | | 315 | 311 |  |
|  | GACTGCTTGCTCCCTGTTGACCAT | – | | 317 | 320 |  |
|  | ACTGCTTGCTCCCTGTTGACCATT | – | | 318 | 313 |  |
|  | ACTGCTTGCTCCCTGTTGACCATT | + | | 318 | 313 |  |
|  | CTGCTTGCTCCCTGTTGACCATTG | + | | 319 | 315 |  |
|  | TGTTGACCATTGGGTATTCTGAAC | + | | 331 | 334 |  |
|  | ATTGGGTATTCTGAACCATCGAGC | + | | 339 | 345 |  |
|  | TTGGGTATTCTGAACCATCGAGCC | + | | 340 | 337 |  |
|  | GGGTATTCTGAACCATCGAGCCAT | – | | 342 | 337 |  |
|  | GGTATTCTGAACCATCGAGCCATG | + | | 343 | 345 |  |
|  | ATGGCTGCCAAGCTGACTCGCCTC | + | | 364 | 367 |  |
| siRNA (24 nt) | | Transcriptional direction | | T_4_ generation read count | T_5_ generation read count | |
| GCGAACGCCTTGGTGCCACCTTCT | | – | | 398 | 400 | |
| CGAACGCCTTGGTGCCACCTTCTC | | + | | 399 | 395 | |
| AACGCCTTGGTGCCACCTTCTCCT | | + | | 401 | 404 | |
| AACGCCTTGGTGCCACCTTCTCCT | | – | | 401 | 404 | |
| ACGCCTTGGTGCCACCTTCTCCTC | | + | | 402 | 399 | |
| CGCCTTGGTGCCACCTTCTCCTCC | | + | | 403 | 398 | |
| GCCTTGGTGCCACCTTCTCCTCCC | | + | | 404 | 406 | |
| CCTTGGTGCCACCTTCTCCTCCCA | | + | | 405 | 401 | |
| TTGGTGCCACCTTCTCCTCCCATC | | + | | 407 | 410 | |
| GGTGCCACCTTCTCCTCCCATCCC | | + | | 409 | 404 | |
| GTGCCACCTTCTCCTCCCATCCCA | | + | | 410 | 412 | |
| GCCACCTTCTCCTCCCATCCCAAT | | – | | 412 | 408 | |
| TGGCTGCCAAGCTGACTCGCCTCC | | + | | 365 | 360 | |
| GCCAAGCTGACTCGCCTCCACAGT | | – | | 370 | 372 | |
| CAAGCTGACTCGCCTCCACAGTCT | | – | | 372 | 368 | |
| AAGCTGACTCGCCTCCACAGTCTT | | – | | 373 | 376 | |
| AGCTGACTCGCCTCCACAGTCTTC | | – | | 374 | 380 | |
| TGACTCGCCTCCACAGTCTTCGCG | | + | | 377 | 374 | |
| ACTCGCCTCCACAGTCTTCGCGAA | | – | | 379 | 374 | |
| CTCGCCTCCACAGTCTTCGCGAAC | | – | | 380 | 382 | |
| CGCCTCCACAGTCTTCGCGAACGC | | – | | 382 | 378 | |
| GCCTCCACAGTCTTCGCGAACGCC | | – | | 383 | 386 | |
| CCTCCACAGTCTTCGCGAACGCCT | | – | | 384 | 379 | |
| CTCCACAGTCTTCGCGAACGCCTT | | – | | 385 | 387 | |
| TCCACAGTCTTCGCGAACGCCTTG | | + | | 386 | 382 | |
| CACAGTCTTCGCGAACGCCTTGGT | | – | | 388 | 391 | |
| ACAGTCTTCGCGAACGCCTTGGTG | | + | | 389 | 395 | |
| AGTCTTCGCGAACGCCTTGGTGCC | | + | | 391 | 388 | |
| GTCTTCGCGAACGCCTTGGTGCCA | | + | | 392 | 387 | |
| CTTCGCGAACGCCTTGGTGCCACC | | + | | 394 | 396 | |
| TTCGCGAACGCCTTGGTGCCACCT | | + | | 395 | 391 | |
| TCGCGAACGCCTTGGTGCCACCTT | | + | | 396 | 399 | |
| TCGCGAACGCCTTGGTGCCACCTT | | – | | 396 | 399 | |
| ATGTTCACCAGGGCAAGGGAATGC | | + | | 461 | 457 | |
| siRNA (24 nt) | | Transcriptional direction | | T_4_ generation read count | T_5_ generation read count |  |
| GTTCACCAGGGCAAGGGAATGCTT | | – | | 463 | 469 |  |
| ACCACTTCCAGCCCCCCAAGAACT | | – | | 149 | 145 |  |
| ACTTCCAGCCCCCCAAGAACTGGA | | + | | 152 | 155 |  |
| AGCCCCCCAAGAACTGGATCAATG | | + | | 158 | 164 |  |
| CCCCCCAAGAACTGGATCAATGAT | | – | | 160 | 157 |  |
| GATCAATGATCCCAACGCGCCCAT | | – | | 174 | 169 |  |
| ATCAATGATCCCAACGCGCCCATG | | + | | 175 | 177 |  |
| ATGATCCCAACGCGCCCATGTACT | | + | | 179 | 175 |  |
| ATCCCAACGCGCCCATGTACTACA | | + | | 182 | 185 |  |
| ACCTTCTCCTCCCATCCCAATGAA | | + | | 415 | 418 |  |
| CCTTCTCCTCCCATCCCAATGAAC | | – | | 416 | 422 |  |
| CTTCTCCTCCCATCCCAATGAACT | | – | | 417 | 414 |  |
| CTCCTCCCATCCCAATGAACTGAT | | – | | 420 | 415 |  |
| TCCTCCCATCCCAATGAACTGATA | | – | | 421 | 423 |  |
| ATCCCAATGAACTGATAGCACTCT | | – | | 428 | 424 |  |
| ATCCCAATGAACTGATAGCACTCT | | + | | 428 | 424 |  |
| TCCCAATGAACTGATAGCACTCTT | | + | | 429 | 424 |  |
| TCCCAATGAACTGATAGCACTCTT | | – | | 429 | 424 |  |
| CCAATGAACTGATAGCACTCTTTT | | – | | 431 | 427 |  |
| AATGAACTGATAGCACTCTTTTCC | | + | | 433 | 436 |  |
| TGAACTGATAGCACTCTTTTCCAG | | + | | 435 | 441 |  |
| ACTGATAGCACTCTTTTCCAGGTA | | + | | 438 | 440 |  |
| TGTTCACCAGGGCAAGGGAATGCT | | – | | 462 | 465 |  |

**Table S3. Analysis of mechanical sorting accuracy after introgression of the SSMM system into five maize inbred lines**

The data shown in the table were obtained from the field investigation conducted at the Sanya experimental site in Hainan, China, in March 2026. After mechanical sorting, 4759, 4565, 4783, 4621, and 4896 candidate normal kernels from LM4, X24621, JS005, Y98-8, and JD60, respectively, were sown and evaluated for plant phenotypes. Among the resulting plants, 15, 23, 31, 24, and 38 abnormal purple plants were observed in the five lines, corresponding to mechanical sorting accuracies of 99.685%, 99.496%, 99.352%, 99.481%, and 99.224%, respectively. The total sample size refers to the total number of plants that were successfully established in the field after sowing the candidate normal kernels obtained by mechanical sorting and subsequently reached the tasseling stage; plants that failed to grow normally, died, or did not tassel were excluded from the analysis. At the pollen-shedding stage, plant fertility was evaluated based on pollen production from the tassels. Plants with tassels that shed visible pollen were classified as fertile, whereas plants with tassels that produced no pollen, failed to shed pollen, or showed anther abortion were classified as sterile.

| **Sample** | **The number of total plants** | **The number of purple plants** | **The ratio of purple plants** | **The ratio of fertile plants in purple plants** | **The ratio of fertile plants in green plants** |
| --- | --- | --- | --- | --- | --- |
| LM4 | 4759 | 15 | 0.315% | 100% | 0% |
| X24621 | 4565 | 23 | 0.504% | 100% | 0% |
| JS005 | 4783 | 31 | 0.648% | 100% | 0% |
| Y98-8 | 4621 | 24 | 0.519% | 100% | 0% |
| JD60 | 4896 | 38 | 0.776% | 100% | 0% |

**Table S4. Primer information**

| **Primer name** | **Primer sequence（5’**–**3’）** |
| --- | --- |
| *Mn1*-qpcr-f | GGACATCTCGAACGGCAAGA |
| *Mn1*-qpcr-r | AAGTAACGAGCCGACACAGC |
| *Sh1*-qpcr-f | GGAGTTTGATGCCCTGTTTGA |
| *Sh1*-qpcr-r | GCACAAAGTTGTGTCCATCCA |
| *Lc*-qpcr-f | AGTGCCCGAGCTCCAGCC |
| *Lc*-qpcr-r | GCCTCTATGTCGTCCATGCCATTAT |
| *zSSIIb*-f | CGGTGGATGCTAAGGCTGATG |
| *zSSIIb*-r | AAAGGGCCAGGTTCATTATCCTC |
| SSMM-ZTF | TCTACAAAGGCTTCAGGTGT |
| SSMM-ZTR | GCATCGGTAACATGAGCAAA |
| *Ms45*-f | ACACTAGACATGGCTGGTCCTCG |
| *Ms45*-r | GATTGAATCCTGTTGCCGGTCTT |
| *Mn1*/*Sh1*-RNAi-f | AGTCGCTCGAACGGACTAA |
| *Mn1*/*Sh1*-RNAi-r | TGGGCTCGGTTTGATTTAGC |
| *Lc*-f | GACCACGTAGTACCACTCGGTGTC |
| *Lc*-r | CATTGCCCAGCTATCTGTCACTT |
| *Bar*-f | ATCGAGACAAGCACGGTCAA |
| *Bar*-r | TCAACACATGAGCGAAACCCT |

**Table S5. One-way ANOVA results**

One-way ANOVA results for 100-kernel weight **(Figure S5D).**

| **ANOVA table** | **F (DFn, DFd)** | ***P* value** |
| --- | --- | --- |
| Treatment (between columns) | F (3, 116) = 445.9 | *P* < 0.0001 |

One-way ANOVA results for agronomic traits **(Figure S7).**

|  | **ANOVA table** | **F (DFn, DFd)** | ***P* value** |
| --- | --- | --- | --- |
| JS005（growth period） | Treatment (between columns) | F (2, 297) = 1.075 | 0.3425 |
| LM4（growth period） | Treatment (between columns) | F (2, 297) = 0.7494 | 0.4735 |
| Y98-8（growth period） | Treatment (between columns) | F (2, 297) = 2.586 | 0.0770 |
| JD60（growth period） | Treatment (between columns) | F (2, 297) = 0.2569 | 0.7736 |
| X24621（growth period） | Treatment (between columns) | F (2, 297) = 1.672 | 0.1896 |
| JS005（plant height） | Treatment (between columns) | F (2, 297) = 2.305 | 0.1016 |
| LM4（plant height） | Treatment (between columns) | F (2, 297) = 0.5165 | 0.5972 |
| Y98-8（plant height） | Treatment (between columns) | F (2, 297) = 1.307 | 0.2722 |
| JD60（plant height） | Treatment (between columns) | F (2, 297) = 2.706 | 0.0685 |
| X24621（plant height） | Treatment (between columns) | F (2, 297) = 0.1928 | 0.8247 |
| JS005（ear height） | Treatment (between columns) | F (2, 297) = 1.438 | 0.2390 |
| LM4（ear height） | Treatment (between columns) | F (2, 297) = 0.1455 | 0.8646 |
| Y98-8（ear height） | Treatment (between columns) | F (2, 297) = 1.152 | 0.3175 |
| JD60（ear height） | Treatment (between columns) | F (2, 297) = 0.8299 | 0.4371 |
| X24621（ear height） | Treatment (between columns) | F (2, 297) = 1.332 | 0.2656 |

**Materials and methods**

***Agrobacterium*-mediated genetic transformation of maize and transgene determination**

Maize genetic transformation was performed using immature embryos of the maize inbred line B73 as explants. The process involved co-cultivation, selection, and regeneration to obtain transgenic seedlings. Immature embryos (1.2–1.5 mm) at 9–11 DAP were selected based on their developmental stage. Excised embryos were immersed in an *Agrobacterium tumefaciens* suspension and then transferred to co-cultivation medium and incubated for 3 days at 20°C in the dark. Subsequently, embryos were transferred to resting medium and then incubated for 7 days at 28°C in darkness. All embryos were transferred to selection medium and incubated for 2 weeks to induce callus formation. The resulting calli were placed on regeneration medium to promote shoot and root development. Regenerated plantlets with three or four leaves were acclimatized in a greenhouse, after which putative T_0_ transgenic plants were identified using rapid lateral flow assays for *bar*-encoded phosphinothricin acetyltransferase protein.

**Anthocyanin content measurement**

At the V7 stage of transgenic maize and non-transgenic control maize, a distal 10-cm segment of the seventh expanded leaf was collected (three biological replicates and three technical replicates). Weigh the sample (weighing range: 0.2000g–3.0000 g) and place it in a 100 mL Erlenmeyer flask. Add 40 mL of hydrochloric acid-ethanol solution, then place the flask in an ultrasonic cleaner for extraction at 30°C for 30 min. Transfer the extract to a 100 mL volumetric flask. Add another 20 mL of hydrochloric acid-ethanol mixed solution to the Erlenmeyer flask, perform ultrasonic extraction at 30°C for 10 min, and transfer the extract to the same volumetric flask. Repeat this extraction step once more, combine all extracts into the volumetric flask, and dilute to the marked volume with the hydrochloric acid-ethanol mixed solution, followed by thorough mixing. A 10 mL aliquot of the mixed extract was centrifuged at 10,000 rpm for 10 min, and collect the supernatant. Transfer the supernatant into a cuvette, use the hydrochloric acid-ethanol mixed solution as the blank reference, and measure the absorbance at a wavelength of 520 nm. Anthocyanin content was measured and compared using Student’s *t*-test.

**Transformant-specific PCR Assay**

We conducted transformant-specific detection of the junction regions at both ends of SSMM by designing a pair of specific primers (SSMM-ZTF/SSMM-ZTR). In this primer pair, one primer anneals to the T-DNA sequence and the other to the flanking genomic sequence, while the other primer target the flanking sequences of the exogenous insert in the genome. Genomic DNA was extracted from maintainer lines using the CTAB method and subsequently subjected to PCR amplification. Genotyping of the abnormal plant samples was performed by amplification using event-specific primers (SSMM-ZTF and SSMM-ZTR). Genotyping of the abnormal endosperm samples was performed by multiplex PCR in a single reaction using transformant-specific primers (SSMM-ZTF and SSMM-ZTR) and internal control primers (*zSSIIb*-f and *zSSIIb*-r). For each endosperm sample, a small portion of endosperm was excised before sowing and used for DNA extraction.

**Quantitative real-time PCR**

The introduction of the *Mn1*/*Sh1*-RNAi cassette induced silencing of the target genes *Mn1* and *Sh1*. To assess their transcript levels in transgenic and recipient materials, kernel samples at 20 DAP and leaf samples at the V7–V8 stage were collected from the SSMM transgenic line and non-transgenic control B73. Total RNA was extracted using the TRIzol, followed by first-strand cDNA synthesis with the HiScript III 1st Strand cDNA Synthesis Kit (+gDNA wiper). Quantitative PCR was performed using the Hieff UNICON® Universal Blue qPCR SYBR Green Master Mix. Fluorescence signals were monitored, and threshold cycle (Ct) values were determined during the exponential PCR phase. Relative quantification of the target sequences (*Mn1*, *Sh1*, *Lc*) was calculated using the 2^-ΔΔCt^ method, and significant differences were determined by Student’s *t*-test.

**Pollen staining**

Using maize as the experimental material, fresh pollen was collected and placed on glass slides for pollen viability analysis. One to two drops of I_2_-KI staining solution were added, and the samples were covered with coverslips. Images were captured using a Nikon SMZ1270 microscope.

**Fertility investigation**

At the pollen-shedding stage, plant fertility was evaluated based on pollen production from the tassels. Plants with tassels that shed visible pollen were classified as fertile, whereas plants with tassels that produced no pollen, failed to shed pollen, or showed anther abortion were classified as sterile. Figure 1J and 1L，the total surveyed population refers to the total number of plants that grew normally in the field and successfully reached the tasseling stage; plants that failed to grow normally, died, or did not tassel were excluded from the analysis.

**Germination assay**

For each replicate, 100 *SSMM*^+^ seeds and 100 non-transgenic control seeds were soaked in water for 6 h and then placed on germination paper. Seeds were germinated at 28°C for 7 days. Germination was assessed after 7 days, and the germination rate was calculated as follows: germination rate (%) = (number of germinated seeds/100) × 100%. Three biological replicates were performed for each group (n = 3). The germination rate was calculated from three biological replicates, and significant differences were statistically analyzed using Student’s *t*-test.

**Field phenotypic investigation**

For field phenotypic investigation, 100 plants each of non-transgenic B73 and the transgenic maintainer line were subjected to field surveys, where growth period, plant height, and ear height were recorded. Significant differences in these phenotypic traits were evaluated using Tukey’s test. After introgressing the SSMM system into five distinct maize inbred lines, phenotypic identification was performed on 100 plants from each of the recurrent parent, transgenic maintainer line, and non-transgenic male-sterile line under each genetic background. Significant differences in growth period, plant height, and ear height among the three genotypes were assessed using One-way ANOVA, followed by Tukey’s post hoc test for multiple comparisons to identify pairwise differences among genotypes.

**Mechanical seed sorting**

Seed sorting was performed using a 5XFS-10C air-screen cleaner and a 5XZ-8Y pneumatic specific-gravity separator (Hebei Julite Sorting Technology Co., Ltd., China). The air-screen cleaner utilized wind force for screening. By selecting precision punched screens of appropriate specifications according to the kernel size, it removed impurities. The number of screen layers was adjusted to classify the products into large and small kernels. The pneumatic specific-gravity separator worked on the principle of gravity segregation under aerodynamic and frictional forces, efficiently eliminating low-density impurities.

**Statistical analysis**

All statistical analyses and figure preparation were conducted using GraphPad Prism 9.5.
